# A Roland for an Oliver

**Published:** 1890-03

**Authors:** 


ARTICLE II.

### A ROLAND FOR AN OLIVER.

### DR. SHEPARD'S DEFT PARRY OF A THRUST AT HIS HEAD—HE DEFENDS HIS OWN ACTIONS AND THOSE OF THE BOARD OF REGISTRATION IN DENTISTRY— BRANDS THE CHARGES AS A COVERT ATTEMPT TO PREVENT HIS RE-APPOINTMENT.

Dr. L. D. Shepard, upon whom, as chief executive officer of the Massachusetts board of registration in dentistry, the charge made against that body by several dentists of this city, outlined exclusively in Thursday's *Herald*, are intended to fall with crushing weight, seems to have been too long a target for criticism to be easily moved by such attacks. Far from being disturbed by the assertions that he had been guilty of permitting fradulent registrations under the dental laws, of opening the ranks of the profession to state prison convicts and of favoring college graduates in examinations, contrary to the act under which the board exists, he expressed himself as being glad that the matter had come up, as it would serve as an opportunity to give the public an idea of how much good the dental laws are and how well they have been administered by the board of which he is president. He says he has been enthusiastic in his efforts to improve the condition of the profession as far as the laws would permit, and has unsparingly given of his valuable time to this end.

Referring to the charges, in conversation with a *Herald* reporter recently, he said he thought they had been pretty effectually answered before they had been made in the three annual reports of the board since its organization in April 1887. In the first report, dated Dec. 30, 1887, appears this, bearing directly on the charge of fraudulent registration: "The board was undecided what construction should be placed upon the phrase in sec. 3, 'who is at that time engaged in the practice of dentistry in this state'. The board decided to interpret that phrase liberally, and so have in nearly every case granted certificates of registration to those who sent to the secretary the properly filled out and sworn to affidavits. There have been a number of such certificates issued which the board might have withheld if it had thought it wise to go back of a sworn statement. The board decided that such action could not be taken by it without the fullest investigation and most positive proof. It therefore considered it more prudent to leave such investigation for future judical action, to be brought by any one who felt aggrieved."

In the leaflet accompanying this report, which was placed in the hands of every registered dentist, were these

SUGGESTIONS TO THE PROFESSION, relative to their duties in assisting in carrying into effect the purposes of the law: "Your attention is called to the list of registered dentists. There are, doubtless, mistakes in names, degrees, etc. Any one noticing such mistakes is requested to inform the secretary. You will also carefully scrutinize the list, to note those who are registered, who are not legally entitled to be, and those who have failed to register. In the case of the former, it will be remembered that each person who has registered has made and sworn to an affidavit that he was in practice in this state on the 1st day of April, 1887. It is also to be noted that the law especially provides that the certificates of registration 'shall be prima facia evidence' only 'of the right of the holder to practice dentistry in Massachusetts,' and hence can be revoked by a court if proved to have been obtained unjustly, *i. e.*, fraudulently. By the law the board is charged with certain duties, but the prosecuting of offenders is not mentioned among them. Every citizen has an equal interest in upholding law, and every dentist should show his interest in progress, by seeing that offenders in his vicinity comply with the law. The scope and thoroughness of the examinations is left to the discretion of the board, and it is considered by the board right and proper that the history, education and experience of the applicant should be taken into account; also that evidence of graduation from a reputable dental college should cause a variation in the examination."

"In an address delivered by me before the New England Dental Society, in October, 1887," said Dr. Shepard, after having read over the above paragraphs. "I said: 'It is a cardinal principle in political economy that industry is the great preventor of poverty and crime, and that there should be a great caution in legislation to interfere as little as possible with the freedom of people to earn their living in any honorable way that each may select. Most civilized states, however, have adopted the principle that it is well and proper for the state to exercise a paternal care over the people in respect to health. A man engaged in an honorable occupation, though his attainments are few, his skill of a low order and his work poor in quality, has a vested right in that occupation by which he earns his daily bread, which the

STATE HAS NO RIGHT TO TAKE FROM HIM.

It may take his property by right of eminent domain by giving him a fair money equivalent, if the general good demands it; but the state cannot forbid him to continue in an occupation which is honest and honorable, and in itself not injurious to the public good. But it is competent for the state to legislate for the future, to decide what conditions and acquirements and skill are requisite to enter in the future upon a given occupation. The state also can set in operation machinery for the execution of such laws and penalties for violation. It is one of the fundamental principles of all such legislation that what are called ex post facto to laws are unjust and unconstitutional. State after state enacted laws to regulate dental practice, until Massachusetts, usually the foremost in wise legislation, was becoming the dumping ground for the refuse of other states. Dental legislation was in the air, and the contagion finally spread here, so that the law at present in force was enacted and wisely approved by his Excellency Gov. Ames. The state society and the profession throughout the state were largely indifferent, and did but little to aid the cause. Whatever may have been the motives of the dentist who was chiefly instrumental in securing the enactment, I have never failed in any place, and at any time, to give him freely the credit which is his due. But little known and and almost alone, he went about the work which others had neglected, and secured the result in which we all rejoice.

It is the same dentist, Lewis W. Foss, to whom I referred in such complimentary terms, who is the chief instigator of the attack upon me in the *Herald*. That his head should have been turned by his success as a lobbyist is not surprising, and that his disappointment should be great at the want of appreciation shown by his being ignored by the Governor in appointing the five members of the board, notwithstanding his earnest efforts for appointment, is also not surprising. It is a fact, also, that he did not accept the result gracefully, but has, during the past three years, been a self appointed and officious advisor to the board and laid plans to entrap it, and, in revenge for the snubbing that he thinks he has received, has proclaimed quite often and publicly his intention 'to go for the scalp of Dr. Shepard' upon the expiration of his term of office. The motive for his attack lies in this and this alone. It is entirely personal, and should have little interest for the public, and with the exception of stating his motive for this attack, I will not descend to any

PERSONALITIES IN REPLY.

"For the proper execution of any law it is desirable to get men of age and experience, and such men, if successful, find their time worth something. If they give it to the state at a paltry $5 a day, they do so at a great personal pecuniary loss. The 50 cent fee for registration did not give funds enough to pay the expense of a smelling committee to go over the whole state and find out who had committed perjury in falsely swearing that they were practising dentistry on April 1, 1887. The charge is made that there was a lack of vigilance on the part of the board in giving out blank affidavits. The fact is that such blanks were furnished freely, and properly so, to any one who requested, and that several hundred were given out that were never used. It was a necessary part of the work in securing registration.

"From the phraseology of sec, 3 of the act, that 'it shall be the duty of every person who is at that time engaged in the practice of dentistry in this state to cause his or her name, residence and place of business, to be registered with said board.' it will be seen that no account is taken of fitness of character, knowledge, skill, method of doing business, whether honest or infamous, freeman or convict. It is simply a question of fact, and the board, in granting a certificate to Sawtell upon his sworn statement, and with a full knowledge of his crime, had no right to disobey the other clause in the same section : 'Every person engaged in the practice of dentistry within this commonwealth at the time of the passage of this act, and who shall so register with said board as a practitioner of dentistry shall receive a certificate to that effect, and may continue to practise without incurring any of the liabilities or penalties provided in this act."

"It is not true, as claimed in the *Herald* article, that the manifest intent of the law was to restrict the practice of dentistry in Massachusetts solely to those who are competent by practice and theoretical education and by moral qualities, as the act makes no reference at all to these qualifications, but intended to continue in practice every one, good, bad and indifferent, as it must do, who was in practice on its enactment, or be unconstitutional.

THE ELEVATION OF THE PROFESSION

was to come by restricting, in the future, licenses to those competent, and it is especially noticeable in the law that the examination of future applicants shall be upon dentistry and dental surgery, no authority being conferred by the law to investigate character. While this may be a serious defect, it is not competent for the board to inject it into the law which they have taken their oaths to carry out. Because dental colleges require certificates of moral character as they should, before conferring their degrees, it does not follow that a board, acting under a law of the commonwealth, can make this demand without authorization of law.

"If Dr. Dennett knew, as is said in the article, that a colored man who was employed as a servant in his office in the year that the registration law was passed made an affidavit that he was a practitioner of dentistry, although he had had no actual experience, and that one justice of the peace to whom he applied to execute the affidavit, knowing his experience, refused to accommodate him, it was Dr. Dennett's duty to the public as a patriotic citizen to make complaint that said colored man had committed perjury so that his certificate, which the law says is only prima facie evidence of right, should be cancelled by the court, and truth and justice vindicated by punishment for perjury. The same remark applies to the negro employed as a servant in Dr. Methol's office. The board would be assuming grave responsibilities in deciding the very delicate question of treating as perjurers any persons who, in their individual opinions, fell below an imaginary line about which, in the nature of things, there must be great diversity of opinion separating rightful practitioners of dentistry from those claiming and swearing they were such, but who might be proved by judicial investigation, to have usurped the title. A case in point is that of Ralph Gorman, a Lenox druggist, who had for years served the people of that place as an extractor of teeth. He did not claim to be a dentist in the full sense of the word, and asked for a license to extract teeth. The law does not admit of a partial license, and the board was called upon to decide whether it would be proper to register this man. He was the only person in the place who made any pretensions of attending to teeth, and his patients included many of the most prominent of the Lenox inhabitants. The board asked themselves whether, if a license were withheld from Mr. Gorman, in view of the fact that, in case it were, the people of Lenox would be deprived of the services of a teeth extractor who had given them good service for years, and would be compelled to travel several miles to a neighboring town or to import a dentist, any jury of 12 men would uphold them? They concluded that full justice to all persons interested required that Mr. Gorman be registered."

In regard to the charge of discrimination in examinations, Dr. Shepard considered the personal criticisms as trivial and unworthy of reply, but claimed that the law leaves to the board the determination of the scope and thoroughness of the examination, and that it is competent for the board to vary the scope and thoroughness of the examination for different applicants—for instance, a certain scope and thoroughness for graduates of the Massachusetts colleges, in respect to each of whose graduates it has had intimate and positive knowledge without any examination; another scope and thoroughness for graduates of colleges from other states, and still another for those who have not had college advantages, and for those who are total strangers. "The intent of the law," he continued, "is to protect the people from the attempt of the incompetent to injure the people of the commonwealth, and not to inaugurate a system of objectionable inquisition. The basis for the decision whether the applicant possesses the 'requisite qualifications,' is not necessarily confined to the formal answering of questions on a certain day, but may be founded, as was the case of Dr. Allen of New York, who graduated in 1860 and had been in the habit for 10 years of attending to a certain family that he came to Boston to operate upon occasionally, and who were attended to by Dr. Allen in my office. In the examination of Dr. Allen, I did not ask him a single question, as I had positive knowledge for years and years, from personal observation of his work and many discussions on professional topics, that he was a highly educated, eminent and skillful practitioner.

"In the examination, last December, of Dr. William Barker of Providence, who is an ex-president of several dental societies, a graduate of and for some years the professor of operative dentistry in the Boston Dental College, and whose operations I had frequently seen, a similar course was pursued. In such cases it is sufficient that the examiner can certify in accordance with his oath that he has found the applicant to possess the 'requisite qualifications.'

"The animus for the personal feeling of Dr. Dennett against me dates from the time when the absurd claims of the Dennett Dental Narbolt Company were exposed, and the profession freed from an unjust royalty upon the patenting of an old remedy, and Dr. Dennett was expelled by a nearly unanimous vote, for unprofessional conduct, from the Massachusetts Dental Society, both of which results were largely due to my devotion to the interests of the profession.

"The fact is that the dental law is working a very great good to the people of the state, as well as to the profession. Year by year the incompetents who were licensed when the law went into effect, are dying off, and year by year about 40 thoroughly competent men are added to the list of practitioners. If the law, when first passed, had been administered in a harsh, injudicious and objectionable manner, the opposition to it would, doubtless, have secured its early repeal. A few cases of unjust or unwise decisions, such as depriving a man of the right to continue a practice, humble though it was, which he was prosecuting when the law was passed, because the educated and skillful men thought him a quack or poor operator, would have brought sufficient discredit upon the law to convince the Legislature that it was not designed for the good of the people."

DR. WHITE DENIES IT.

To-day, after the foregoing interview was in type, Dr. Shepard received a letter which he has embodied in the following:

*To the Editor of the Herald*: Unexpectedly and unsolicited I received by this morning's mail the inclosed letter. It has reference to the following extract from the *Herald* article: "Another fault found with the board is that, contrary to the expressed intent of the law, they discriminate in their examinations in favor of graduates of dental colleges. It is said that many of the latter are not examined at all. One young man, Dr. White, a graduate of the Harvard dental school, is quoted as saying that when he went before Dr. Shepard to qualify he was turned off with a wave of the hand and the remark: "Oh, you're all right. I know all about you. You'll do."

I have not seen Dr. White or had any communication with him since the days last July when he operated before the board and was examined. His spontaneous branding as false the above statement comes to me quite opportunely. It shows the unscrupulousness of the calumniators of the board and its president. Dr. White objects to being used as a bearer of false witness. While his letter is a private one, I feel justified in making it public, as he says he has already requested the *Herald* to deny the truth of the charge. Dr. White's letter is as follows:

BRIDGEWATER, Mass., March 2, 1890.

Dr. L. D. Shepard, 100 Boylston street—Dear Sir: It was with regret that I noticed in the Boston *Herald* of Feb. 27 an article in which my name is used in connection with a statement said to have been made by me, and which I have requested the *Herald* to deny, and to which I refer you.

Respectfully yours,

J. R. WHITE.

I am sorry to take up so much of your space, but I feel that the board is the wronged party.

L. D. SHEPARD.

